# RETRACTED: Bellomo et al. Effect of *Bifidobacterium bifidum* Supplementation in Newborns Born from Cesarean Section on Atopy, Respiratory Tract Infections, and Dyspeptic Syndromes: A Multicenter, Randomized, and Controlled Clinical Trial. *Microorganisms* 2024, *12*, 1093

**DOI:** 10.3390/microorganisms13092097

**Published:** 2025-09-09

**Authors:** Anna Rita Bellomo, Giulia Rotondi, Prudenza Rago, Silvia Bloise, Luigi Di Ruzza, Annamaria Zingoni, Susanna Di Valerio, Eliana Valzano, Francesco Di Pierro, Massimiliano Cazzaniga, Alexander Bertuccioli, Luigina Guasti, Nicola Zerbinati, Riccardo Lubrano

**Affiliations:** 1 Dipartimento Materno Infantile e di Scienze Urologiche, Sapienza Università di Roma, UOC di Pediatria e Neonatologia-Polo Pontino, 04100 Latina, Italy; annaritabellomo62@gmail.com (A.R.B.); prudenzarago@gmail.com (P.R.); silvia.bloise1989@gmail.com (S.B.); riccardo.lubrano@uniroma1.it (R.L.); 2 Pediatric Surgery Unit, Gaslini Children Hospital and Research Institute, 16147 Genoa, Italy; giuliarotondi1996@gmail.com; 3 UOC Pediatria e Nido, Ospedale S.S. Trinità, 03039 Sora, Italy; ludir62@gmail.com; 4 UOC Pediatria e Neonatologia, Ospedale G.B. Grassi, 00122 Ostia, Italy; a.m.zingoni@libero.it; 5 UOC Neonatologia e Terapia Intensiva Neonatale, Ospedale S. Spirito, 65124 Pescara, Italy; susanna.divalerio@ausl.pe.it (S.D.V.); neonatologia.pe@ausl.pe.it (E.V.); 6 Scientific & Research Department, Velleja Research, 20125 Milan, Italy; maxcazzaniga66@gmail.com; 7 Department of Medicine and Surgery, University of Insubria, 21100 Varese, Italy; luigina.guasti@uninsubria.it (L.G.); nicola.zerbinati@uninsubria.it (N.Z.); 8 Department of Biomolecular Sciences, University of Urbino Carlo Bo, 61029 Urbino, Italy; alexander.bertuccioli@uniurb.it

The journal retracts the article, “Effect of *Bifidobacterium bifidum* Supplementation in Newborns Born from Cesarean Section on Atopy, Respiratory Tract Infections, and Dyspeptic Syndromes: A Multicenter, Randomized, and Controlled Clinical Trial” [1].

Following publication, concerns were brought to the attention of the publisher related to ethical oversight [1].

Adhering to our standard procedure, an investigation was conducted by the Editorial Office and the Editorial Board, with input from a representative of the Sapienza University of Rome, that confirmed that this study does not fully adhere to MDPI’s Research Involving Human Subjects policy (https://www.mdpi.com/ethics#_bookmark9). As a result, the Editorial Board has decided to retract this article [1], in line with MDPI’s retraction policy (https://www.mdpi.com/ethics#_bookmark30).

This retraction was approved by the Editor-in-Chief of the journal *Microorganisms*.

The authors, Anna Rita Bellomo, Giulia Rotondi, Prudenza Rago, Silvia Bloise, and Riccardo Lubrano, agree to this retraction. The remaining authors did not provide a comment on this decision.

## References

[1] Bellomo A.R., Rotondi G., Rago P., Bloise S., Di Ruzza L., Zingoni A., Di Valerio S., Valzano E., Di Pierro F., Cazzaniga M. (2024). RETRACTED: Effect of *Bifidobacterium bifidum* Supplementation in Newborns Born from Cesarean Section on Atopy, Respiratory Tract Infections, and Dyspeptic Syndromes: A Multicenter, Randomized, and Controlled Clinical Trial. Microorganisms.

